# Postoperative intubation time is associated with acute kidney injury in cardiac surgical patients

**DOI:** 10.1186/s13054-014-0547-4

**Published:** 2014-10-03

**Authors:** Matthias Heringlake, Yvonne Nowak, Julika Schön, Jens Trautmann, Astrid Ellen Berggreen, Efstratios I Charitos, Hauke Paarmann

**Affiliations:** Department of Anesthesiology and Intensive Care Medicine, University of Lübeck, Ratzeburger Allee 160, 23538 Lübeck, Germany; Department of Cardiac and Thoracic Vascular Surgery, University of Lübeck, Ratzeburger Allee 160, 23538 Lübeck, Germany

## Abstract

**Introduction:**

Acute kidney injury (AKI) is a frequent complication after cardiac surgery and is associated with a poor prognosis. Mechanical ventilation is an important risk factor for developing AKI in critically ill patients. Ventilation with high tidal volumes has been associated with postoperative organ dysfunction in cardiac surgical patients. No data are available about the effects of the duration of postoperative respiratory support in the immediate postoperative period on the incidence of AKI in patients after cardiac surgery.

**Method:**

We performed a secondary analysis of 584 elective cardiac surgical patients enrolled in an observational trial on the association between preoperative cerebral oxygen saturation and postoperative organ dysfunction and analyzed the incidence of AKI in patients with different times to extubation. The latter variable was graded in 4 h intervals (if below 16 h) or equal to or greater than 16 h. AKI was staged according to the AKI Network criteria.

**Results:**

Overall, 165 (28.3%) patients developed AKI (any stage), 43 (7.4%) patients needed renal replacement therapy. Patients developing AKI had a significantly (*P* <0.001) lower renal perfusion pressure (RPP) in the first 8 hours after surgery (57.4 mmHg (95% CI: 56.0 to 59.0 mmHg)) than patients with a postoperatively preserved renal function (60.5 mmHg ((95% CI: 59.9 to 61.4 mmHg). The rate of AKI increased from 17.0% in patients extubated within 4 h postoperatively to 62.3% in patients ventilated for more than 16 h (*P* <0.001). Multivariate logistic regression analysis of variables significantly associated with AKI in the univariate analysis revealed that the time to the first extubation (OR: 1.024/hour, 95% CI: 1.011 to 1.044/hour; *P* <0.001) and RPP (OR: 0.963/mmHg; 95% CI: 0.934 to 0.992; *P* <0.001) were independently associated with AKI.

**Conclusion:**

Without taking into account potentially unmeasured confounders, these findings are suggestive that the duration of postoperative positive pressure ventilation is an important and previously unrecognized risk factor for AKI in cardiac surgical patients, independent from low RPP as an established AKI trigger, and that even a moderate delay of extubation increases AKI risk. If replicated independently, these findings may have relevant implications for clinical care and for further studies aiming at the prevention of cardiac surgery associated AKI.

**Electronic supplementary material:**

The online version of this article (doi:10.1186/s13054-014-0547-4) contains supplementary material, which is available to authorized users.

## Introduction

Cardiac surgery-associated acute kidney injury (CSA-AKI) is an important complication and an independent mortality factor in patients undergoing cardiac surgery [1] and even minor deteriorations in postoperative renal function have been associated with an increased mortality risk [2].

The pathophysiological factors mediating CSA-AKI may be summarized as a complex interplay between a decrease in renal blood flow (induced either directly by changes in systemic perfusion or indirectly by activation of the renin-angiotensin system and/or the renal sympathetic nervous system), hyperglycemia and increased inflammation as well as intrinsic and extrinsic toxins (that is myoglobin and hemoglobin, radiocontrast agents) (for overview see: [1,3,4]). No therapeutic concept to prevent or ameliorate this complication has been proved to be successful in adequately powered trials [3,4].

Cardiac surgical procedures are typically performed during general anesthesia and most patients are mechanically ventilated in the postoperative period until normothermia and hemodynamic stability have been achieved. Interestingly, the duration of postoperative positive pressure ventilation has been largely ignored as a factor potentially mediating CSA-AKI in almost all interventional studies as well as in scientific reviews of this field, despite the fact that it has long been known that especially mechanical ventilation with positive end-expiratory pressure (PEEP), but also continuous positive airway pressure (CPAP) breathing may have profound effects on renal function [5,6].

Very recently, van den Akker and coworkers have shown that mechanical ventilation for more than 24 h is associated with a threefold increase in the risk for developing acute kidney injury (AKI) in critically ill patients [7]. However, only one small study including cardiac surgical patients was included in this meta-analysis.

Lellouche and coworkers analyzed the effects of ventilation with low (<10 ml/kg), traditional (10 to 12 ml/kg) or high (>12 ml/kg) tidal volumes immediately after cardiac surgery on the incidence of postoperative organ dysfunction and observed a clear association between non-protective ventilation and organ dysfunction including AKI. However, and despite patients with low tidal volumes being extubated earlier after surgery than patients with traditional or high, that is injurious tidal volumes, the authors did not address the effects of short-term ventilation (below 24 h) on organ dysfunction in this trial [8].

The present study thus explores the influence of the duration of continuous postoperative positive pressure ventilation within the immediate postoperative period on the incidence of CSA-AKI in a heterogeneous cohort of cardiac surgical patients.

## Materials and methods

The present study is a secondary analysis of data from a large observational trial on the relationship between preoperative cerebral oxygen saturation (ScO2) determined by near-infrared spectroscopy (NIRS) and postoperative organ dysfunction. This data set has already been used for other analyses and the details of recruitment as well as consenting have been presented elsewhere [9,10]. Following approval from the local ethics committee (Ethikkommission der Universität zu Lübeck, Lübeck, Germany), all patients scheduled for cardiac surgery at the University of Lübeck from 1 April 2009 to 29 December 2009 were screened for participation in this trial. Written informed consent was obtained preoperatively.

**Figure 1:**
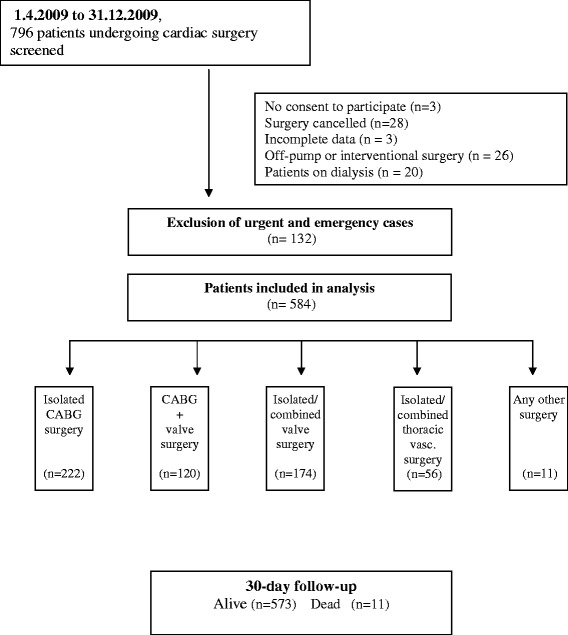
**Trial population and design.**

The only exclusion criterion for the observational trial was an age of less than 18 years. The present analysis was restricted to elective patients and additionally excluded patients preoperatively treated with dialysis (Figure 1).

Anesthesiological, surgical, and intensive care treatment followed standardized algorithms established at the Department of Anesthesiology and the Department of Thoracic Vascular and Cardiac Surgery of the University of Lübeck and have also been presented in detail elsewhere [9,10]. Hemodynamic treatment was partially adapted from a German guideline for the hemodynamic treatment of cardiac surgical patients [11]. Accordingly, intra- and postoperative fluid therapy was performed with balanced Ringer’s solution, 6% hydroxyethyl starch 130, and gelatinesuccinate at the discretion of the attending physicians. Starting in July 2009, all patients were additionally treated perioperatively with a continuous infusion of 4 mmol/kg sodium bicarbonate [9].

Surgery was performed during mild hypothermia using antegrade blood cardioplegia. After surgery, patients were transferred to the ICU while being sedated with a combination of remifentanil and propofol adjusted to their clinical needs. In absence of contraindications (glucose-6-phosphate dehydrogenase deficiency, porphyria), a continuous infusion of metamizole 3 g/24 h was started. After normal body temperature had been achieved, propofol was stopped, analgesics (pethidine and/or piritramide) were applied, and the remifentanil infusion was continuously reduced. Further piritramide was applied according to patients’ analgesic needs.

Postoperative ventilation was performed with biphasic intermittent positive airway pressure (BIPAP) and pressure support ventilation (PSV) with a minimum PEEP of 5 mbar and inspiratory pressures/pressure support-adjusted to achieve a tidal volume around 6 to 8 ml/kg. Postoperative adjustments in ventilatory support as well as weaning from the respirator and extubation were performed at the discretion of the attending physicians of the ICU.

The severity of postoperative kidney dysfunction was quantified by the Acute Kidney Injury Network (AKIN) criteria [12] from perioperative changes in plasma creatinine (relative increase in relation to the preoperative baseline) and urine flow (within the first 24 h after surgery).

### Statistical analysis

Analyses were performed with MedCalc 12.7.7.0 for Windows and R version 3.1.0 [13]. For comparisons of the descriptive characteristics, simple statistical tests (chi-square test, Wilcoxon, Mann-Whitney test, Kruskal-Wallis test) were used as appropriate. A *P* <0.05 was considered to indicate statistical significance. If not specified otherwise, data are presented as median and the 95% confidence interval (CI) of the median.

Patients were grouped according to the postoperative time until first extubation into five groups: <4 h; ≥4 h to <8 h; ≥8 h to <12 h; ≥12 h to <16 h; ≥16 h. The cutoff value of 16 h for ‘prolonged’ ventilation was chosen due to the clinical observation that a cardiac surgical patient ventilated for more than 16 h after surgery usually cannot be discharged routinely on the morning of the first postoperative day.

Renal perfusion pressure (RPP) was calculated as the difference between mean arterial (MAP) and central venous pressure (CVP).

Univariate analyses were used to identify variables with significant statistical influence on the development of postoperative AKI. The following variables that showed a highly significant statistical difference (*P* <0.001) in the univariate analyses were entered a stepwise backward logistic regression model: age; Euroscore; preoperative plasma creatinine level; ScO2_minox_; type of surgery; duration of surgery; duration of cardiopulmonary bypass (CPB); lowest hematocrit during CPB; intra- and postoperative use of inotropic/vasoactive agents (noradrenaline, dobutamine, vasopressine); cumulative use of blood products; mean RPP within the first 8 hours after ICU admission, time to first extubation (hours), and reintubation. RPP was used as a surrogate parameter of MAP and CVP to avoid overfitting of the model.

## Results

### Risk factors and treatments in patients with and without AKI

Overall, 165 (28.3%) patients developed AKI of any stage (AKI stage 1: 85 (14.6%), AKI stage 2: 7 (1.2%), AKI stage 3: 73 (12.5%)). Forty-three (7.4%) patients needed renal replacement therapy (RRT). Of these patients, 33 (20%), 7 (4.2%), and 1 (0.6%) fulfilled the AKI urine output criteria for the AKI stages 1 to 3, respectively, while the other 75.1% primarily met the AKI creatinine criteria or were treated with RRT upon clinical indications (inadequate response to diuretics, acidosis, and so on).

With the exception of 10 patients, in which RRT was started later than 48 h postoperatively, (range 49 h to 139 h after surgery), the majority of patients developed AKI in the immediate period after surgery (median time to the start of RRT: 26.5 h (95% CI: 14 h to 40 h).

Patient demographics, cardiovascular risk factors, and additive Euroscore were significantly different in patients with and without postoperative AKI (Table 1). Additionally, surgical core markers (duration of surgery and duration of CPB) pointed to a significantly higher operative risk. Patients with AKI were treated more frequently and with higher doses of inotropes and vasopressors, needed more blood products and showed a trend toward a higher use of synthetic colloids. Specific analysis of the doses of hydroxyethyl starch and gelatine showed that patients with AKI were treated with a significantly higher amount of gelatine (no AKI: 1,500 (1,500 to 2,000) ml, AKI: 2,000 (1,500 to 2,000) ml; *P* =0.009).

**Table 1:** **Demographics, perioperative treatments, and outcomes in patients with or without acute kidney injury**

	**No AKI ** **N =419**	**AKI ** **N =165**	**Significance**
**Age (years)**	67 (65 to 68)	72 (71 to 73)	*P* <0.001
**Male/female (%)**	272/147 (64.9/35.1)	108/57 (65.5/34.5)	*P* =0.98
**Height (cm)**	172 (170 to 173)	170 (168 to 172)	*P* =0.02
**Weight (kg)**	81 (80 to 84)	80 (78 to 83)	*P* =0.62
**Additive Euroscore**	4 (4 to 5)	6 (5 to 7)	*P* <0.001
**Preoperative creatinine (μmol/l)**	79 (77 to 82)	87 (82 to 94)	*P* <0.001
**Preoperative eGFR** ^**§**^ ** (ml/min/1.73 m2)**	83.9 (80.5 to 86.2)	72.6 (67.6 to 78.4)	*P* <0.001
**Preoperative diuretics**			
**Y/N**	227/192	103/62	*P* =0.065
**(%)**	54.2/ 45.8	62.4/37.6	
**ScO2**_**air**_ **(%)**	63 (63 to 64)	61 (60 to 62)	*P* <0.001
**ScO2**_**ox**_ **(%)**	68 (67 to 69)	67 (65 to 68)	*P* =0.005
**ScO2**_**minox**_ **(%)**	62 (61 to 62)	59 (57 to 61)	*P* <0.001
**Preoperative LVEF**			
- **good**	315 (75.1)	122 (73.9)	*P* =0.63
- **moderate**	88 (21)	38 (23)	
- **poor**	16 (3.8)	4 (2.4)	
**CABG**			
- **no**	176 (42)	43 (26.1)	*P* <0.001
- **yes**	243 (58)	122 (73.9)	
**Duration of CPB ** **(min)**	110 (104 to 116)	128 (118 to 140)	*P* <0.001
**Duration of surgery**	245 (236 to 253)	272 (265 to 288)	*P* <0.001
**Minimal hematocrit ** **(%)**	25 (25 to 26)	25 (24 to 25)	*P* =0.002
**Minimal temperature during CBP ** **(°C)**	32 (32 to 32)	32 (32 to 32)	*P* =0.48
**Blood products ** **(units)**	2 (1 to 2)	3 (2 to 4)	*P* <0.001
**Cristalloids** **(ml)**	4560 (4510 to 4640)	4580 (4450 to 4710)	*P* =0.84
**Colloids ** **(ml)**	2000 (2000 to 2500)	2500 (2000 to 2500)	*P* =0.08
**Drainage loss ** **(ml)**	800 (800 to 850)	950 (900 to 1000)	*P* <0.001
**Postoperative urine flow ** **(ml/24 h)**	2930 (2869 to 3050)	2450 (2198 to 2650)	*P* <0.001
**Postoperative diuretics**			
**Y/N**	381/38	145/20	*P* =0.444
**(%)**	90.9%/9.1%	87.8%/12.2%	
**Maximal postoperative lactate ** **(mmol/l)**	1.9 (1.7 to 1.9)	2.4 (2.2 to 2.7)	*P* <0.001
**MAP baseline ** **(%)**	83 (82 to 85)	85 (82 to 89)	*P* =0.32
**MAP mean IOP ** **(mmHg)**	71.7 (71.0 to 72.3)	70.7 (69.5 to 71.7)	*P* =0.11
**CVP mean IOP ** **(mmHg)**	10.0 (9.3 to 10.3)	10.7 (10.0 to 11.5)	*P* =0.006
**ScvO2 mean IOP ** **(%)**	81.5 (80.6 to 82.0)	80.9 (79.2 to 82.7)	*P* =0.34
**SvO2 mean IOP** ^* ^ **(%)**	79.5 (78.5 to 80.7)	79.3 (77.6 to 80.2)	*P* =0.74
**PAP mean IOP** ^*^ **(mmHg)**	28.3 (23.5 to 26.2)	26.9 (24.4 to 29.6)	*P* =0.01
**CI mean IOP** ^*^ **(l/min/m2)**	2.9 (2.8 to 3.1)	1.8 (2.6 to 3.0)	*P* =0.07
**MAP mean POP ** **(mmHg)**	75.2 (74.4 to 76.0)	72.6 (71.6 to 74.2)	*P* <0.001
**CVP mean POP ** **(mmHg)**	14.7 (14.3 to 14.9)	15.2 (14.8 to 16.2)	*P* <0.001
**ScvO2 mean POP ** **(%)**	70.6 (69.5 to 71.7)	69.3 (66.4 to 71.3)	*P* =0.09
**SvO2 mean POP** ^* ^ **(%)**	68.4 (67.1 to 69.5)	68.5 (66.5 to 70.4)	*P* =0.86
**PAP mean POP** ^*^ **(mmHg)**	28.3 (27.6 to 29.2)	30.4 (29.0 to 32.2)	*P* =0.03
**RPP mean POP ** **(mmHg)**	60.5 (59.9 to 61.4)	57.4 (56.0 to 59.0)	*P* <0.001
**CI mean POP** ^*^ **(l/min/m2)**	3.2 (3.0 to 3.3)	3.0 (2.8 to 3.1)	*P* =0.006
**Dobutamine - IOP ** **(no. of pts. (%))**			*P* =0.03
**0**	195 (46.5)	60 (36.4)	
**<15 mg/h**	93 (22.2)	37 (22.4)	
**15 - 30 mg/h**	115 (27.4)	54 (32.7)	
**>30 mg/h**	16 (3.8)	14 (8.5)	
**Dobutamine - POP ** **(no. of pts. (%))**			*P* <0.001
** 0**	159 (37.9)	40 (24.2)	
**<15 mg/h**	137 (32.7)	47 (28.5)	
**15 - 30 mg/h**	107 (25.5)	57 (34.5)	
**>30 mg/h**	16 (3.8)	21 (12.7)	
**PDE - III - IOP** **(no. of pts. (%))**			*P* =0.001
**0**	258 (61.6)	77 (46.7)	
**Low dose**	28 (6.7)	7 (4.2)	
**Moderate dose**	124 (29.6)	74 (44.8)	
**High dose**	10 (2.4)	7 (4.2)	
**PDE III-I - POP ** **(no. of pts. (%))**			*P* <0.001
** 0**	216 (51.6)	55 (33.3)	
**Low dose**	41 (9.8)	19 (11.5)	
**Moderate dose**	145 (34.6)	79 (47.9)	
**High dose**	16 (3.8)	12 (7.3)	
**Noradrenaline - IOP ** **(no. of pts. (%))**			*P* =0.054
**0**	51 (12.2)	11 (6.7)	
**<0.3 mg/h**	192 (45.8)	69 (41.8)	
**0.3 - 0.6 mg/h**	130 (31)	57 (34.5)	
**<0.6 mg/h**	46 (10.9)	28 (16.9)	
**Noradrenaline - POP ** **(no. of pts. (%))**			*P* <0.001
**0**	72 (17.2)	18 (10.9)	
**<0.3 mg/h**	205 (48.9)	57 (34.5)	
**0.3 - 0.6 mg/h**	94 (22.4)	47 (28.5)	
**<0.6 mg/h**	48 (11.5)	43 (26.1)	
**Vasopressine - IOP ** **(no. of pts. (%))**			*P* =0.016
**0**	393 (93.8)	142 (86.1)	
**<3 U/h**	17 (4.1)	16 (9.7)	
**3 - 6 Uh**	8 (1.9)	7 (4.2)	
**>6 U/h**	1 (0.2)	0 (0)	
**Vasopressine - POP ** **(no. of pts. (%))**			*P* <0.001
**0**	384 (91.6)	137 (83)	
**<3 U/h**	31 (7.4)	17 (10.3)	
**3 - 6 Uh**	3 (0.7)	7 (4.2)	
**> 6 U/h**	1 (0.2)	4 (2.4)	
**Time to first extubation (h)**	7 (6 to 7)	8 (7 to 9)	*P* <0.001
**Reintubation**			*P* <0.001
** No**	409 (97.6)	133 (80.6)	
** Yes**	9 (2.1)	32 (19.4)	
**Total ventilation time ** **(h)**	7 (6 to 7)	9 (7.4 to 11)	*P* <0.001
**Duration of ICU treatment ** **(h)**	23 (22 to 23)	66 (38 to 81)	*P* <0.001
**Duration of HDU treatment ** **(h)**	70 (65 to 77)	124 (108 to 144)	*P* <0.001
**Mortality 30 d ** **(no. of pts. (%))**	1 (0.2)	10 (6.1)	*P* <0.001

Continuous data are given as median and 95% confidence interval of the median; categorical data as absolute number with percentages in parenthesis. ^**§**^Estimated glomerular filtration rate determined by the modifications of diet in renal disease equation; ^*^data derived from patients monitored with pulmonary artery catheter. AKI: acute kidney injury; eGFR: estimated glomerular filtration rate; ScO2: cerebral oxygen saturation determined by near-infrared spectroscopy (ScO2_air_: bihemispheric mean values during room air; ScO2_ox_: bihemispheric mean values during oxygen supplementation; ScO2_minox_: lowest value of either side during oxygen supplementation); LVEF: left ventricular ejection fraction; CABG: coronary artery bypass grafting; CPB: cardiopulmonary bypass; IOP: intraoperatively; POP: postoperatively; MAP: mean arterial pressure; CVP: central venous pressure; PAP: mean pulmonary arterial pressure; ScvO2: central venous oxygen saturation; SvO2: mixed venous oxygen saturation; CI: cardiac index; RPP: renal perfusion pressure (MAP - CVP); PDE-III: phosphodiesterase III inhibitors (enoximone or milrinone), ICU: intensive care unit; HDU: high dependency unit. The amount of fluids, blood products (packed red cells and fresh frozen plasma) represents the volumes or units applied during surgery and within the first day on the ICU.

Analysis of intra- and postoperative hemodynamics showed minor, but at several time points statistically significant differences in mean arterial and central venous pressures, central venous oxygen saturation (ScvO2) and, in invasively monitored patients, differences in mean pulmonary artery pressure (PAP) and cardiac index (CI) between patients with postoperative preserved renal function and patients with AKI (Table S1 in Additional file 1 and Table 1). Average renal perfusion pressure during the first postoperative 8 h was significantly lower in patients with AKI in comparison with patients without this complication (Table 1).

### Time to extubation and renal outcomes

Median time to the first extubation was 7 h (6.8 to 7.0 h). Forty-seven patients (8.1%) were reintubated for at least one time. Total postoperative ventilation time in reintubated patients was 190 h (109 to 365 h). Seventeen patients were treated with tracheostomy for long-term respiratory support.

**Figure 2:**
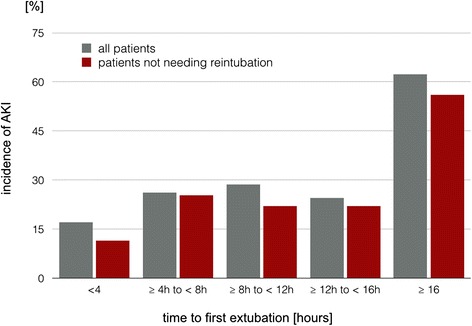
**The incidence of acute kidney injury (AKI) in patients after cardiac surgery according to the time to the first extubation in the total cohort (all patients) and after excluding patients needing reintubation.**

The rate of AKI increased from 17.0% in patients extubated within 4 h postoperatively to 62.3% in patients ventilated for equal or more than 16 h (*P* <0.001) in the total cohort (Figure 2). Exclusion of the 47 patients needing reintubation revealed a lower incidence of AKI (24.6%), but a comparable pattern, with an AKI rate of 12.4% in patients with less than 4 h to first extubation and 53.8% in patients with prolonged (≥16 h) ventilator support (*P* <0.001).

Potential risk factors for longer mechanical ventilation after cardiothoracic surgery in the groups with different extubation times are summarized in Table 2, showing that - for the total cohort - patients that were ventilated longer were older, had a higher Euroscore, a lower estimated glomerular filtration rate (eGFR), a longer duration of surgery, a higher postoperative drainage loss, a higher use of blood products, and were postoperatively treated with higher doses of noradrenaline and dobutamine.

**Table 2:** **Demographics, risk factors, and treatments in patients with different time to extubation after cardiac surgery**

**Time to first extubation**	**(1) ** **≤4 h ** **n =94**	**(2)** **>4 h to ≤8 h ** **n =287**	**(3) ** **>8 h to ≤12 h ** **n =105**	**(4) ** **>12 h to ≤16 h ** **n =45**	**(5) ** **n >16 ** **n =53**	**Significance ** **(within group differences)**	**Significance ** **(between different times to extubation)**
**Age ** **(years)**	63 (58 to 68)	68 (66 to 70)	71 (68 to 73)	68 (65 to 73)	71 (67 to 74)	*P* <0.001	(1) vs. (2) (3) (4) (5)
							(2) vs. (1) (3)
							(3) vs. (1) (2)
							(4) vs. (1)
							(5) vs. (1)
**Additive Euroscore**	4 (3 to 5)	5 (4 to 5)	6 (5 to 6)	5 (4 to 6)	7 (6 to 9)	*P* <0.001	(1) vs. (2) (3) (4) (5)
							(2) vs. (1) (3) (5)
							(3) vs. (1) (2)
							(4) vs. (1) (5)
							(5) vs. (1) (2) (4)
**eGFR (ml/min/1.73 m2)**	86.2 (81.5 to 90.4)	80.4 (78.0 to 85.2)	74.6 (71.4 to 81.2)	84.1 (76.9 to 89.1)	67.6 (57.3 to 90.3)	*P* =0.02	(1) vs. (2) (3) (5)
							(2) vs.(1)
							(3) vs. (1)
							(5) vs. (1)
**Duration of surgery (min)**	222 (205 to 240)	252 (244 to 260)	260 (246 to 276)	263 (248 to 280)	291 (270 to 385)	*P* <0.001	(1) vs. (2) (3) (4) (5)
							(2) vs. (1) (5)
							(3) vs. (1) (5)
							(4) vs. (1) (5)
							(5) vs. (1) (2) (3) (4)
**Drainage loss ** **(ml)**	800 (700 to 900)	800 (800 to 900)	900 (850 to 1000)	900 (745 to 941)	1000 (773 to 1205)	*P* =0.02	(1) vs. (3) (5)
							(2) vs. (3) (5)
							(3) vs. (1) (2)
							(5) vs. (1) (2)
**Blood products (unit)**	1 (0 to 2)	2 (1 to 2)	2 (2 to 3)	2 (2 to 4)	7 (6 to 9)	*P* <0.001	(1) vs. (2) (3) (4) (5)
							(2) vs. (1) (3) (5)
							(3) vs. (1) (2) (5)
							(4) vs. (1) (5)
							(5) vs. (1) (2) (3) (4)
**MAP POP ** **(mmHg)**	76.5 (73.8 to 77.7)	74.4 (73.7 to 75.1)	73.6 (72.4 to 75.1)	76.4 (75.3 to 78.3)	74.0 (71.4 to 75.9)	*P* =0.02	(1) vs. (2) (3) (5)
							(2) vs. (1)
							(3) vs. (1) (4)
							(4) vs. (3)
							(5) vs. (1)
**CVP POP (mmHg)**	13.9 (13.2 to 14.7)	14.4 (14.0 to 14.8)	15.2 (14.9 to 15.9)	15.6 (14.6 to 16.1)	16.9 (15.6 to 18.0)	*P* <0.001	(1) vs. (3) (4) (5)
							(2) vs. (3) (4) (5)
							(3) vs. (1) (2) (5)
							(4) vs. (1) (2)
							(5) vs. (1) (2) (3)
**RPP POP (mmHg)**	62.4 (60.0 to 65.1)	60.1 (59.5 to 61.3)	57.9 (56.8 to 59.6)	60.6 (58.3 to 63.4)	57.8 (53.6 to 59.5)	*P* <0.001	(1) vs. (3) (5)
							(2) vs. (3) (5)
							(3) vs. (1) (2)
							(4) vs. (5)
							(5) vs. (1) (2) (4)
**Dobutamine - POP**						*P* <0.001	
**0**	39 (41.5%)	108 (37.6%)	26 (13.3%)	12 (26.7%)	14 (26.4%)		
**<15 mg/h**	28 (29.8%)	94 (32.8%)	32 (16.4%)	14 (31.1%)	16 (30.2%)		
**15 - 30 mg/h**	23 (24.5%)	72 (25.1%)	41 (21%)	17 (37.8%)	12 (22.6%)		
**>30 mg/h**	4 (4.2%)	14 (4.9%)	6 (3.1%)	2 (4.4%)	11 (20.8%)		
**Noradrenaline - POP**						*P* <0.001	
**0**	18 (19.1%)	57 (19.9%)	9 (4.6%)	3 (6.7%)	3 (5.7%)		
**<0.3 mg/h**	50 (53.2%)	136 (47.4%)	44 (22.6%)	23 (51.1%)	9 (17%)		
**0.3 - 0.6 mg/h**	18 (19.1%)	68 (23.7%)	33 (16.9%)	13 (28.9%)	9 (17%)		
**>0.6 mg/h**	8 (8.5%)	26 (9.1%)	19 (9.7%)	6 (13.3%)	32 (60.4%)		
**AKI stage**						*P* <0.001	
**0**	78 (83%)	212 (73.9%)	75 (71.5%)	34 (75.5%)	20 (37.7%)		
**1**	8 (8.5%)	45 (15.7%)	18 (17,1%)	8 (17.8%)	6 (11.3%)		
**2**	1 (1.1%)	5 (1.7%)	0 (0%)	0 (0%)	1 (1.9%)		
**3**	7 (7.4%)	25 (8.7%)	12 (11.4%)	3 (6.7%)	26 (49.1%)		

Differences in variables strongly associated with acute kidney injury in univariate analysis in groups of cardiac surgical patients stratified according to the times of first extubation after surgery. Data are given as median and 95% confidence interval of the median or absolute numbers with percentages in parenthesis. ScO2_ox_: preoperative oxygen-supplemented cerebral oxygen saturation determined by near-infrared spectroscopy. eGFR: estimated glomerular filtration rate by the Modifications of Diet in Renal Disease (MDRD) formula; POP: postoperative; MAP: average mean arterial pressure within 8 h after surgery; CVP: average central venous pressure within 8 h after surgery; RPP: average renal perfusion pressure (MAP - CVP) within 8 h after surgery; AKI: acute kidney injury. Kruskal-Wallis and chi-square test.

Analyses of hemodynamics within the first 8 h after surgery in patients with different times to extubation revealed that patients ventilated for more than 16 h had a lower postoperative MAP, a higher CVP and, consecutively, a lower RPP than patients ventilated for a shorter periods of time. There was no significant differences in RPP between patients ventilated for ≤4 h and >4 h to ≤8 h after surgery (Table 2).

Logistic regression in the total cohort showed that time to the first extubation was a predictor of AKI taking into account variables significantly associated with AKI in the univariate analysis and the need for reintubation. In the total cohort, the multivariate logistic regression showed that the duration of ventilation (odds ratio (OR): 1.024/hour, 95% CI: 1.011 to 1.044/hour; *P* <0.001), age (OR: 1.035/year, 95% CI: 1.015 to 1.057/hour; *P* <0.001), duration of surgery (OR: 1.003, 95% CI: 1.001 to 1.006; *P* =0.02), RPP (OR: 0.963/mmHg; 95% CI: 0.934 to 0.992/mmHg; *P* <0.001), and reintubation (OR: 5.27; 95% CI: 2.545 to 11.499; *P* <0.001) were independently associated with AKI.

**Figure 3:**
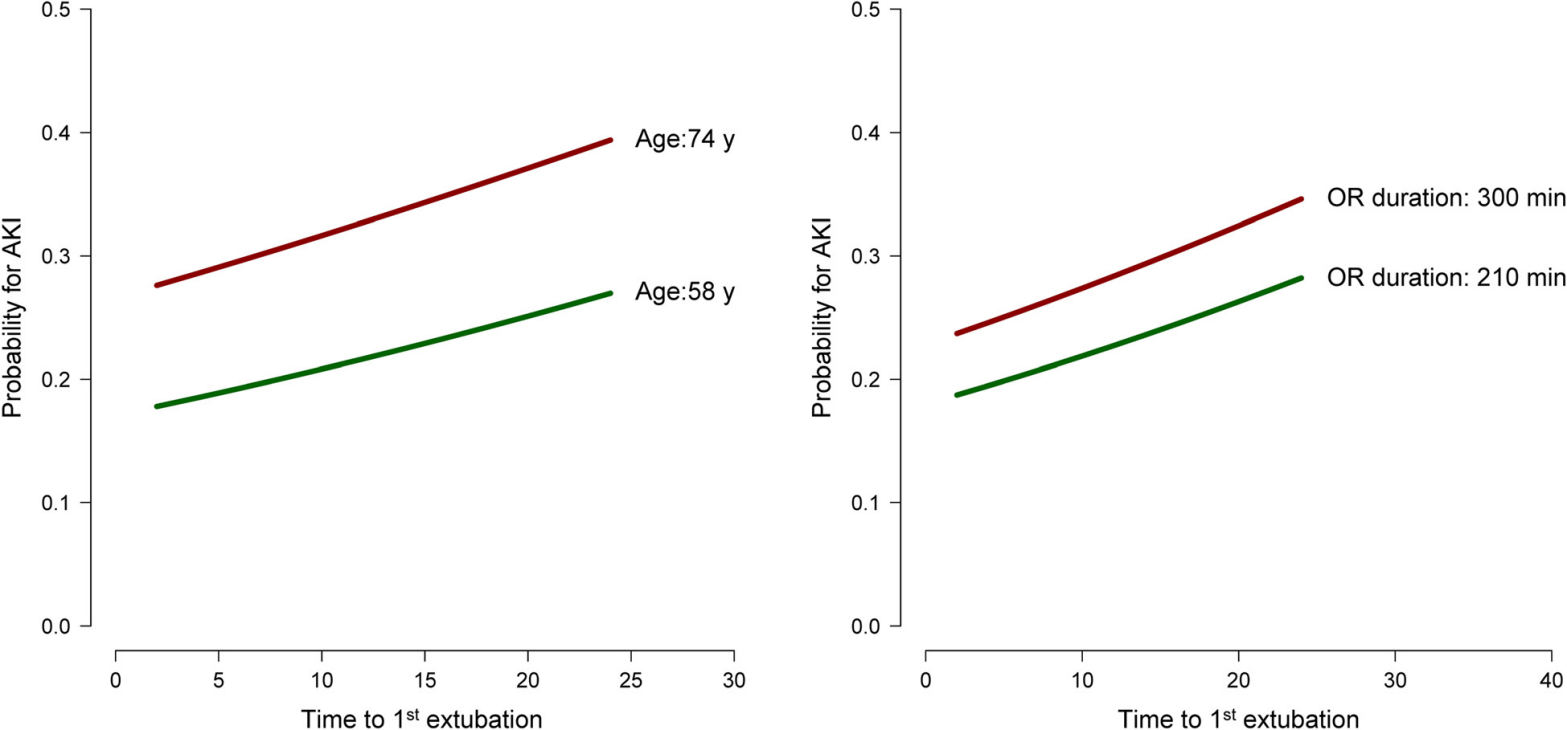
**Graphical depiction of the results of the multivariate logistic regression model in the total cohort using prototypical input values (25th and 75th percentiles of the respective variables).** Intubation time (x-axis) independently increased the probability of developing acute kidney injury (y-axis). **Left:** Duration of surgery 266 min; mean renal perfusion pressure 60 mmHg. **Right:** Age 64 years old, mean renal perfusion pressure 60 mmHg. Intubation time (x-axis) independently increased the probability of developing acute kidney injury (y-axis).

**Figure 4:**
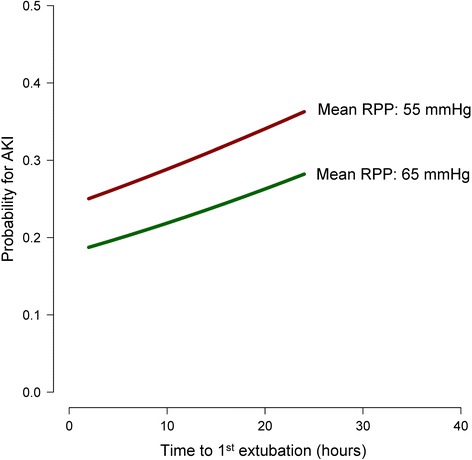
**Graphical depiction of the effect of intubation time and renal perfusion pressure on the probability of developing acute kidney injury (y-axis) for a prototypical patient (age 66 years; duration of surgery 266 min).**

Figures 3 and 4 present the results of the multivariate logistic regression model for prototypical input values (25^th^ and 75^th^ percentile) of the model’s variables.

## Discussion

The present study aims to determine the effects of the duration of postoperative ventilation on the incidence of AKI in a heterogeneous cohort of elective cardiac surgical patients. The results of this analysis are highly suggestive that - among other well-accepted risk factors for AKI like age and renal hypoperfusion - a delay of extubation substantially increases the risk for developing AKI.

AKI is an important complication in patients undergoing cardiac surgery [1,4]. Several mechanisms mediating a perioperative decrease in renal function have been identified within the last years. Among these factors are: exogeneous and endogeneous toxins, anemia, atheroembolic events, systemic and renal inflammation, hyperglycemia, and neurohumoral activation [1,3,4,14]. However, a decrease in RPP and renal blood flow leading to a reduction in oxygen delivery remains a common and important pathway in these patients. The latter mechanism has frequently been attributed to perioperative periods of overt low systemic blood flow, anemia, and/or hypotension [15,16], especially during CPB. It has been largely ignored that almost all patients undergoing cardiac surgery are mechanically ventilated during and after surgery, and that positive pressure ventilation itself impacts renal function via humoral and neurohumoral pathways ultimately leading to a decrease in glomerular filtration rate and renal blood flow [5,6]. It is of note that the complex neurohumoral effects of mechanical ventilation on the kidney are - at least partially - independent from overt changes in hemodynamics and may be difficult to detect using conventional clinical monitoring [17].

Thus, despite the interactions between established mediators of CSA-AKI and mechanical ventilation have not been formally analyzed, it is rather plausible that mechanical ventilation may act synergistically with established AKI - triggers to further reduce renal function in the perioperative setting.

The findings of the present study clearly support this concept: according to the results of the logistic regression analysis every hour of postoperative mechanical ventilation independently increases the odds of developing AKI by a factor of 1.024. It is of note that the time to the first extubation was more important as a risk factor for AKI than conventional risk stratification tools like additive Euroscore [18] and cerebral oxygen saturation [10] and remained statistically significant in the multivariate logistic regression after adjustment for the fact that patients developing AKI also had a lower RPP in the immediate time period after surgery. Interestingly, the average MAP in patients that developed AKI in the postoperative course was in a range that is conventionally regarded as safe, the CVP values were only slightly above normal, and only the calculation of the effective RPP revealed that patients developing AKI suffered from (still numerically moderate) renal hypoperfusion.

Despite the effects of positive airway pressure on kidney function have been the focus of several experimental studies [6], clinical data on this topic from cardiac surgical patients are sparse. Van den Akker and coworkers have shown in a meta-analysis, that mechanical ventilation for more than 24 h is associated with a threefold increase in the risk for developing AKI in critically ill patients [7]. However, only one study in cardiac surgical patients was included in this analysis. Brito and coworkers studied 186 patients during a three-year period and observed, that in the univariate analysis, mechanical ventilation for more than 24 h was a risk factor of AKI (defined as a 50% increase in creatinine or need for dialysis). Probably as an effect of the relatively small sample size, this variable was no longer significant in multivariate analysis [19].

Koning and coworkers analyzed the effects of intermittent positive airway pressure (IPPV) and spontaneous ventilation on cardiac output, renal blood flow, creatinine clearance, and urinary electrolyte excretion and observed decreased renal blood flow and creatinine clearance during IPPV conditions that partially improved during spontaneous ventilation. However, no data on the effects of mechanical ventilation on incidence of AKI were presented in this study [20].

Within the last years, further interactions between mechanical ventilation and AKI have been observed that may also have implications for cardiac surgical patients. A wealth of experimental [21] and an increasing number of clinical studies are suggestive that non-protective ventilation may induce pulmonary biotrauma, inflammation, and subsequent non-pulmonary organ dysfunction (including kidney dysfunction) [22,23]. In line with this, Lellouche and coworkers observed that cardiac surgical patients ventilated with traditional (10 ml/kg) or higher tidal volume are at increased risk for extrapulmonary organ dysfunction as well as prolonged ICU stay and that these complications translate into poor long-term outcomes [8]. And despite some other studies failing to show an effect of the ventilation mode on systemic inflammation [24], a recent meta-analysis - including several studies in cardiac surgical patients - came to the conclusion that protective ventilation with lower volumes may lead to better clinical outcomes [25].

### Limitations

Despite the sound pathophysiological background described above, it has to be acknowledged that patients in the present study who were not extubated immediately after surgery were older, had a higher risk profile, had a lower RPP, and were more frequently treated with vasoactive or inotropic drugs than patients extubated early. However, the results of the logistic regression analysis are clearly indicative that the duration of postoperative positive pressure ventilation is an independent risk factor of AKI, taking into account all the above-mentioned factors. Nonetheless, some uncertainty remains, since it cannot definitively be ruled out that the adverse effects of longer ventilation after surgery may be mediated by unrecognized or undetermined confounders like the nephrotoxic effects of synthetic colloids [26] that were more frequently used in patients with AKI or short-term variations in hemodynamics that remained undetectable during hourly measurements.

Unfortunately, we have no data on individual patient ventilation strategies in the present study. Tidal volumes during mechanical ventilation or while breathing spontaneously with pressure support were adjusted by nurses and physicians according to standard hospital practice in the range between 6 and 8 ml/kg. However, it cannot, of course, be completely ruled out that periods of non-protective ventilation may have occurred in these patients and have induced a proinflammatory state amplifying the development of AKI.

With respect to the observational nature of the present study we did not perform a power analysis *a priori* for this specific research question. The relatively high number of events (that is patients developing AKI) strengthens our findings, even if the total sample size was only moderate. However, our findings present only a single-center experience and thus may need to be replicated in a prospective and multicenter fashion.

## Conclusions

In conclusion, the results of the present study suggest that mechanical ventilation is an important trigger for AKI in cardiac surgical patients and that even a moderate delay of extubation after surgery may substantially increase the risk of developing AKI. If replicated independently, this observation may not only have relevant implications for clinical management (supporting the concept of fast-track cardiac anesthesia) but also for AKI trials.

## Key messages

- Acute kidney injury (AKI) is a serious and frequent complication in patients undergoing cardiac surgery.
- Positive pressure ventilation may influence renal hemodynamics via neurohumoral pathways.
- The present observational study shows that the time to the first extubation after cardiac surgery is independently associated with the risk of developing AKI.
- These findings suggest that positive pressure ventilation is an important and previously unrecognized risk factor for AKI in cardiac surgical patients and that even a moderate increase in the duration of postoperative ventilation time may lead to a substantial increase in the risk for developing AKI.

## Additional file

Additional file 1: Table S1. Perioperative hemodynamics in cardiac surgical patients with or without acute kidney injury.

## Abbreviations

AKI: acute kidney injury
AKIN: Acute Kidney Injury Network
BIPAP: biphasic intermittent positive airway pressure
CABG: coronary artery bypass grafting
CI: cardiac index
CPAP: continuous positive airway pressure
CPB: cardiopulmonary bypass
CSA-AKI: cardiac surgery-associated acute kidney injury
CVP: central venous pressure
eGFR: estimated glomerular filtration rate
HDU: high dependency unit
ICU: intensive care unit
IPAP: intermittent positive airway pressure
LVEF: left ventricular ejection fraction
MAP: mean arterial blood pressure
NIRS: near-infrared spectroscopy
PAP: mean pulmonary arterial pressure
PEEP: positive end-expiratory pressure
PSV: pressure support ventilation
RPP: renal perfusion pressure
RRT: renal replacement therapy
ScO2: cerebral oxygen saturation
ScvO2: central venous oxygen saturation
