# Supplementary material for: Postoperative intubation time is associated with acute kidney injury in cardiac surgical patients
Source: Crit Care. 2014 Oct 3;18(5):547. doi: 10.1186/s13054-014-0547-4 (PMC4209080; doi:10.1186/s13054-014-0547-4)
Supplement: Additional file 1: Table S1. — Perioperative hemodynamics in cardiac surgical patients with or without acute kidney injury. [file 13054_2014_547_MOESM1_ESM.doc]

**Additional file 1: Table S1.**

**Perioperative hemodynamics in cardiac surgical patients with or without acute kidney injury.**

|  | **No AKI**  **N = 419** | **AKI**  **N = 165** | **significance** |
| --- | --- | --- | --- |
| **MAP IOP 0**  **[mmHg]** | 83  (82 to 85) | 85  (82 to 89) | P = 0.32 |
| **MAP IOP 1**  **[mmHg]** | 70  (70 to 71) | 70  (69 to 72) | P = 0.49 |
| **MAP IOP 2**  **[mmHg]** | 70  (69 to 71) | 68  (65 to 69) | P = 0.015 |
| **MAP IOP 3**  **[mmHg]** | 73  (72 to 74) | 70  (69 to 72) | P = 0.06 |
| **MAP POP 0**  **[mmHg]** | 77  (75 to 79) | 73  (70 to 75) | P < 0.001 |
| **MAP POP 1**  **[mmHg]** | 76  (75 to 77) | 71  (70 to 73) | P < 0.001 |
| **MAP POP 2**  **[mmHg]** | 73  (72 to 75) | 73  (71 to 74) | P = 0.17 |
| **MAP POP 3**  **[mmHg]** | 74  (72 to 75) | 71  (70 to 74) | P = 0.01 |
| **MAP POP 4**  **[mmHg]** | 74 (73 to 76) | 72  (70 to 74) | 0.13 |
| **MAP POP 5**  **[mmHg]** | 74  (73 to 76) | 71  (70 to 74) | P = 0.02 |
| **MAP POP 6**  **[mmHg]** | 74  (73 to 75) | 72  (70 to 73) | P = 0.006 |
| **MAP POP 7**  **[mmHg]** | 75  (74 to 75) | 72  (71 to 74) | P < 0.001 |
| **MAP POP 8**  **[mmHg]** | 75  (74 to 75) | 71  (70 to 74) | P = 0.002 |
| **Table 1ES cont.** |  |  |  |
| **CVP IOP 0**  **[mmHg]** | 11  (10 to 11) | 12  (11 to 12) | P = 0.026 |
| **CVP IOP 1**  **[mmHg]** | 8  (7 to 8) | 9  (8 to 10) | P < 0.001 |
| **CVP IOP 2**  **[mmHg]** | 11  (10 to 11) | 11  (10 to 12) | P = 0.14 |
| **CVP IOP 3**  **[mmHg]** | 11  (11 to 12) | 12  (12 to 13) | P = 0.035 |
| **CVP POP 0**  **[mmHg]** | 15  (15 to 16) | 15  (15 to 17) | P = 0.28 |
| **CVP POP 1**  **[mmHg]** | 15  (15 to 15) | 16  (15 to 16) | P = 0.13 |
| **CVP POP 2**  **[mmHg]** | 15  (15 to 15) | 16  (15 to 17) | P < 0.001 |
| **CVP POP 3**  **[mmHg]** | 15  (14 to 15) | 16  (15 to 17) | P < 0.001 |
| **CVP POP 4**  **[mmHg]** | 15  (14 to 15) | 16  (15 to 17) | P = 0.001 |
| **CVP POP 5**  **[mmHg]** | 15  (14 to 15) | 16  (14 to 17) | P = 0.006 |
| **CVP POP 6**  **[mmHg]** | 14  (13 to 14) | 16  (14 to 17) | P = 0.008 |
| **CVP POP 7**  **[mmHg]** | 13  (13 to 14) | 15  (15 to 16) | P < 0.001 |
| **CVP POP 8**  **[mmHg]** | 13  (12 to 13) | 15  (14 to 16) | P < 0.001 |
|  |  |  |  |
|  |  |  |  |
|  |  |  |  |
| **Table 1ES cont.** |  |  |  |
| **PAP IOP 0**  **[mmHg]** | 25  (23 to 27) | 28  (25 to 30) | P = 0.15 |
| **PAP IOP 1**  **[mmHg]** | 26  (23 to 27) | 25  (23 to 29) | P = 0.19 |
| **PAP IOP 2**  **[mmHg]** | 25  (23 to 27) | 28  (25 to 32) | P = 0.003 |
| **PAP IOP 3**  **[mmHg]** | 25  (23 to 27) | 28  (26 to 30) | P = 0.02 |
| **PAP POP 0**  **[mmHg]** | 29  (27 to 30) | 30  (28 to 33) | P = 0.13 |
| **PAP POP 1**  **[mmHg]** | 29  (28 to 30) | 30  (28 to 33) | P = 0.31 |
| **PAP POP 2**  **[mmHg]** | 29  (27 to 30) | 32  (30 to 34) | P = 0.007 |
| **PAP POP 3**  **[mmHg]** | 29  (28 to 31) | 30  (29 to 32) | P = 0.10 |
| **PAP POP 4**  **[mmHg]** | 29  (28 to 30) | 31  (29 to 34) | P = 0.04 |
| **PAP POP 5**  **[mmHg]** | 28  (27 to 30) | 30  (28 to 33) | P = 0.10 |
| **PAP POP 6**  **[mmHg]** | 28  (27 to 29) | 29  (27 to 32) | P = 0.23 |
| **PAP POP 7**  **[mmHg]** | 28  (27 to 29) | 29  (27 to 31) | P = 0.22 |
| **PAP POP 8**  **[mmHg]** | 28  (26 to 29) | 28  (27 to 31) | P = 0.22 |
|  |  |  |  |
|  |  |  |  |
|  |  |  |  |
| **Table 1ES cont.** |  |  |  |
| **CI IOP 0**  **[mmHg]** | 2.2  (2.0 to 2.5) | 2.3  (2.0 to 2.7) | P = 0.56 |
| **CI IOP 1**  **[mmHg]** | 2.6  (2.4 to 2.7) | 2.7  (2.5 to 2.9) | P = 0.97 |
| **CI IOP 2**  **[mmHg]** | 3.1  (2.9 to 3.3) | 3.1  (3.0.to 3.3) | P = 0.58 |
| **CI IOP 3**  **[mmHg]** | 3.1  (3.0 to 3.3) | 2.8  (2.7 to 2.9) | P = 0.004 |
| **CI POP 0**  **[mmHg]** | 2.8  (2.7 to 2.9) | 2.6  (2.5 to 2.9) | P = 0.36 |
| **CI POP 1**  **[mmHg]** | 3.0  (2.9 to 3.2) | 2.9  (2.6 to 3.0) | P = 0.36 |
| **CI POP 2**  **[mmHg]** | 3.0  (2.9 to 3.2) | 2.8  (2.7 to 3.1) | P = 0.19 |
| **CI POP 3**  **[mmHg]** | 3.1  (2.9 to 3.2) | 2.8  (2.8 to 3.3) | P = 0.10 |
| **CI POP 4**  **[mmHg]** | 3.2  (3.0 to 3.3) | 3.0  (2.8 to 3.3) | P = 0.13 |
| **CI POP 5**  **[mmHg]** | 3.2  (3.0 to 3.4) | 3.0  (2.7 to 3.1) | P = 0.007 |
| **CI POP 6**  **[mmHg]** | 3.3  (3.1 to 3.4) | 3.2  (3.0 to 3.3) | P = 0.19 |
| **CI POP 7**  **[mmHg]** | 3.3  (3.1 to 3.5) | 3.0  (2.8 to 3.0) | P = 0.003 |
| **CI POP 8**  **[mmHg]** | 3.3  (3.1 to 3.4) | 3.1  (2.8 to 3.3) | P = 0.03 |
|  |  |  |  |
|  |  |  |  |
|  |  |  |  |
| **Table 1ES cont.** |  |  |  |
| **RRP IOP 0**  **[mmHg]** | 73  (70 to 75) | 74  (69 to 77) | P = 0.79 |
| **RRP IOP 1**  **[mmHg]** | 62  (61 to 63) | 62  (60 to 64) | P = 0.65 |
| **RRP IOP 2**  **[mmHg]** | 59  (58 to 60) | 56  (54 to 59) | P = 0.002 |
| **RRP IOP 3**  **[mmHg]** | 61  (60 to 62) | 59  (57 to 61) | P = 0.004 |
| **RRP POP 0**  **[mmHg]** | 61  (60 to 62) | 56  (54 to 60) | P < 0.001 |
| **RRP POP 1**  **[mmHg]** | 61  (60 to 62) | 56  (53 to 58) | P < 0.001 |
| **RRP POP 2**  **[mmHg]** | 58  (57 to 60) | 57  (55 to 58) | P = 0.005 |
| **RRP POP 3**  **[mmHg]** | 59  (58 to 61) | 55  (53 to 56) | P < 0.001 |
| **RRP POP 4**  **[mmHg]** | 60  (58 to 61) | 56  (54 to 59) | P = 0.005 |
| **RRP POP 5**  **[mmHg]** | 61  (58 to 62) | 56  (54 to 58) | P < 0.001 |
| **RRP POP 6**  **[mmHg]** | 61  (59 to 62) | 57  (56 to 59) | P < 0.001 |
| **RRP POP 7**  **[mmHg]** | 61  (60 to 62) | 58  (56 to 59) | P < 0.001 |
| **RRP POP 8**  **[mmHg]** | 62  (61 to 63) | 58  (55 to 61) | P < 0.001 |

Hemodynamic parameters at predefined time points intraoperatively (IOP) and postoperatively (POP) in patients with or without acute kidney injury after cardiac surgery. Data are given as median and 95% confidence interval of the median. MAP: mean arterial pressure; CVP: central venous pressure; PAP: mean pulmonary artery pressure; CI: cardiac index; RPP: average renal perfusion pressure (MAP – CVP). IOP – time points: 0: After induction; 1: before cardiopulmonary bypass (CPB); 2: immediately after CPB; 3: after sternal closure. POP: 0: upon admission to the ICU and hourly for 8 hours (1 to 8). Data for PAP and CI are derived from 224 patients monitored with pulmonary artery catheter. Mann-Whitney test.
